# Treatment of squamous cell carcinoma of the uterine cervix with radiation therapy alone: long-term survival, late complications, and incidence of second cancers

**DOI:** 10.1038/sj.bjc.6604005

**Published:** 2007-09-25

**Authors:** T Ota, N Takeshima, T Tabata, K Hasumi, K Takizawa

**Affiliations:** 1 Department of Gynecology, Cancer Institute Hospital, Ariake 3-10-6, Koutou-ku, Tokyo 135-8550, Japan

**Keywords:** cervical carcinoma, radiation, late complication, second cancer

## Abstract

The objective of this retrospective study was to determine the survival rate, incidence of late complications, and incidence of second cancers when radiation therapy alone is used for carcinoma of the uterine cervix. Between 1971 and 1995, 1495 patients with squamous cell carcinoma of the uterine cervix (stages I–IV) were treated with radiation therapy alone in our hospital. Radiation therapy consisted of a combination of high-dose-rate intracavitary brachytherapy and external beam radiotherapy. The cumulative 5-year survival rates for stages Ib, II, and III/IVa carcinoma were 93.5, 77.0, and 60.3%, respectively, and the 10-year survival rates were 90.9, 74.5, and 56.1%, respectively. Local control rates for stages Ib, II, and III/IVa carcinoma were 92.0, 79.4 and 64.2%, respectively. Eighty-two (5.5%) patients suffered grade III/IV or V (fatal) complications. A second cancer developed in 13 (0.87%) patients. Second cancers were observed most frequently in the rectum (five cases), colon (three cases), and uterine body (two cases). Long-term follow-up data revealed that our method of radiation therapy alone for locally advanced carcinoma of the uterine cervix is effective, with low incidences of late complications and second cancers.

Cervical cancer is one of the most common cancers in women worldwide. Despite worldwide efforts at screening with the aim of detecting cervical cancer in the early stage, many cancers are not discovered until already advanced. Traditionally, radical hysterectomy or radiation therapy alone has been accepted as standard treatment for early-stage invasive cervical cancer, and locally advanced cancer has been treated by radiotherapy alone consisting of a combination of high-dose-rate intracavitary brachytherapy (ICBT) and external beam radiotherapy (EBRT) (Coia *et al*, 1990; Komaki *et al*, 1995; Barillot *et al*, 1997). In the past few years, substantial advances in the management of locally advanced cervical cancer have been reported. Five randomised trials showed improved survival and local control when cisplatin-based chemotherapy was added concurrently to radiation treatment in patients with locally advanced cervical cancer (Keys *et al*, 1999; Morris *et al*, 1999; Rose *et al*, 1999; Whitney *et al*, 1999; Peters *et al*, 2000). This combined modality approach produced an absolute increase in 5-year survival of 12% as compared with radiation therapy alone and resulted in a sudden change in the standard of care for this disease. Now that concurrent treatment with cisplatin-based chemotherapy and radiotherapy is the standard treatment for locally advanced cervical cancer, the main issue in treatment is how chemotherapy is used, not much attention is given to the radiotherapy method.

For more than 30 years, we have treated locally advanced cervical cancer by radiation therapy alone. In this study, we retrospectively analysed 1495 cases of cervical carcinoma treated at our institution from 1971 to 1995 by a unique combination EBRT/ICBT regimen. We were interested in determining the long-term survival rate as well as the incidences of late complications and second cancers.

## METHODS

### Study population

From 1971 through 1995, a total of 1600 new patients with primary invasive cervical carcinoma were treated by radiotherapy alone at the Cancer Institute Hospital, Tokyo, Japan. Of these patients, 1495 (93.4%) had pure squamous cell carcinoma (SCC). The remaining 105 patients with non-SCC were excluded from this study. After an initial clinical examination, all patients underwent a complete staging workup, including a complete blood count, blood chemistry tests, chest radiography, and biopsy of the cervical tumour. Cystoscopy and drip infusion pyelography were performed in all patients; only in suspicious case was sigmoidoscopy performed.

The numbers of patients are shown by cancer stages in Table 1. Stages were determined according to the International Federation of Gynecology and Obstetrics criteria. Patients with distant metastasis (ie stage IVb cervical carcinoma) before treatment and patients treated by chemotherapy before radiation were excluded.

### Treatment schedule

**Figure 1:**
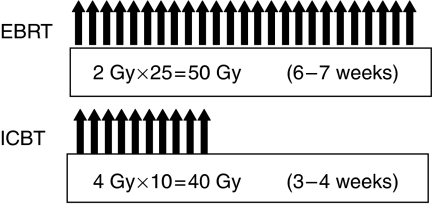
Treatment schedule for radiation therapy.

The radiation treatment schedule is shown in Figure 1. A combination of EBRT and ICBT was used in all patients according to a regimen that is unique to our institution. For all patients, including those with stage III or IV disease, it was planned that EBRT and ICBT would begin simultaneously. However, in 431 patients (28.9%) we were unable to insert the uterine applicator at the time of radiotherapy was initiated because of the tumour bulk, thus, EBRT was started alone without the use of a midline shield until 20 Gy was delivered; ICBT was performed later. In patients who initially received 20 Gy with unshielded EBRT, reduction of one to three fractions of ICBT was considered, depending on the radiation effect. Occasionally, ICBT could not be applied even after EBRT. Indeed, in 13 patients, radiotherapy was achieved with EBRT alone. Overall treatment time ranged from 6 to 7 weeks.

External beam radiotherapy to a total dose of 50 Gy was delivered in 2 Gy fractions per day, 5 days a week, by using an isocentric technique via a pair of parallel opposed anterior and posterior ports and 18-MV X-ray beam. Both pelvic and para-aortic fields were irradiated except in patients with non-bulky stage I disease; only pelvic radiation was performed in these patients. The superior margin of the para-aortic radiation field was the L2–3 intervertebral space, the lateral limit of each pelvic field was 2 cm lateral to the most lateral point of the pelvic wall, and the lower limit was the caudal pole of the obturator foramen. A midline shield was inserted throughout the external radiation period (except in patients in whom EBRT was started before ICBT) regardless of the stage of cervical carcinoma. The midline shield was 4 cm wide at the central axis.

High-dose-rate remote afterloading ICBT was performed with a uterine applicator and two vaginal applicators used with ^60^Co sources (2–4 Ci). Low-dose consecutive brachytherapy was prescribed to all patients. A dose of 4 Gy in one fraction was routinely prescribed to point A, which was located 2 cm superior to the cervical ostium and 2 cm lateral to the central axis of the uterus. Intracavitary brachytherapy was performed with two or three insertions per week, for a total of 10 fractions. When ICBT was applied to a bulky tumour at the start of treatment, the dose of radiation delivered to point A was gradually adjusted in parallel with the reduction in tumour size. Because ICBT was divided into 10 fractions, this process was accomplished without severe morbidity. Orthogonal radiographs were obtained for each insertion, and each treatment session was planned. In the majority of cases, neither anaesthesia nor sedation was necessary during ICBT. EBRT and ICBT were not performed on the same day. When ICBT was performed, EBRT was performed the next day.

### Survival

Survival of each patient was calculated from the date therapy was started to the date of the last follow-up examination. Survival curves were drawn according to the Kaplan–Meier method. The log-rank test was used for univariate analysis. *P*-values of less than 0.05 were considered statistically significant.

Complete response was defined as absence of cancer cells as determined by smear cytology and biopsy of the uterine cervix. Recurrence was defined as reappearance of cancer cells.

### Local control rate

Complete response was defined as no remaining cancer cells according to cytologic and histologic assessment for over 3 months after radiotherapy. Local control was defined as absence of recurrence in the pelvic cavity.

### Late complications

Late rectal and bladder complications and non-rectal gastrointestinal sequelae (small bowel complications) were graded according to the Radiation Therapy Oncology Group (*RTOG*)/the European Organization for Research and Treatment of Cancer (*EORTC*) scoring system (Cox *et al*, 1995).

### Second cancers

Cancer in the radiation field that differed histologically from the primary cancer, as distinguished from recurrence or metastasis from the primary tumour, was considered a second cancer. The person-years proposed by Schoenberg and Myer (1972) was used for statistical analysis. The disease expectancy table used for the present study was the one provided by the Center for Cancer Control and Information Services, National Cancer Center, Japan, which calculated expected ratios from 1995.

### Follow-up

Follow-up examinations were done every 3 months during the first 5 years after treatment and then at 6-month intervals for the next 5 years. All follow-up examinations included pelvic examination with cytologic assessment of the uterine cervix and tumour marker SCC antigen, and identification of late complications. Every 6 months, we obtained a computed tomography scan of the abdomen and a chest X-ray firm. All patients were followed up for more than 10 years after radiation therapy. Our hospital is one of a few institutions that are permitted to access the family registry database. We consulted the district legal affairs bureau for survival information or the cause of the death pertaining to each patient, so our records were complete; no patient was lost to follow-up. Of the total 1495 patients, 1224 (81.9%) were followed up directly at the hospital and 271 (18.1%) were followed up indirectly through the district legal affairs bureau.

## RESULTS

### Study population

Patient numbers by cancer stage of the study are shown in Table 1. Mean age of the patients was 60.6 years (range, 29–93 years). Of the total 1495 patients, the numbers of patients with stage Ib, II, and III/IVa disease were 174 (11.6), 608 (40.7), and 713 (47.7%), respectively.

### Survival

**Figure 2:**
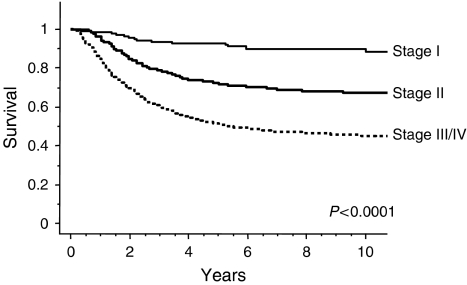
Disease-specific survival per clinical stage for patients with cervical carcinoma treated with radiotherapy alone.

Disease-specific survival curves are shown according to cancer stages in Figure 2. The cumulative 5-year survival rates for stages Ib, II, and III/IVa disease were 93.5, 77.0, and 60.3%, respectively. Ten-year survival rates for stages Ib, II, and III/IVa disease were 90.9, 74.5, and 56.1%, respectively.

### Local control

**Figure 3:**
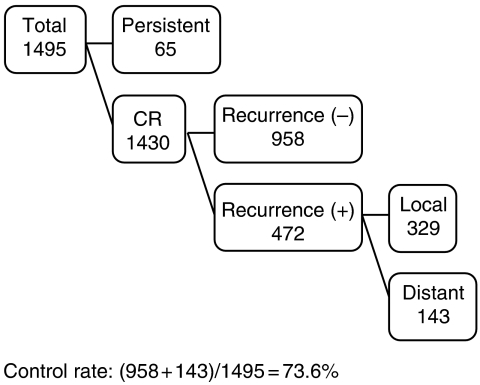
Local control of cervical carcinoma treated by radiotherapy alone.

Local control and recurrence are shown in Figure 3. Of the total 1495 patients, 1430 patients had a complete response, as shown by cytologic and histologic assessment. Of these 1430 patients, 472 patients suffered recurrence (local recurrence, 329 patients; distant metastasis, 143 patients). Thus, the local control rate was 73.6%. Local control rates for stages Ib, II, and III/IVa disease were 92.0, 79.4, and 64.2%, respectively. The most common sites of recurrence were the lung (55 patients) and supraclavicular lymph nodes (35 patients).

### Late complications

Late complications are presented in Table 2. According to the *RTOG/EORTC* scoring system, grade III, IV, or V (fatal) late complications involving the rectum, small bowel, or urinary tract were observed in 97 (6.5%) cases. Sixty-one cases (4.1%) involved stage III disease. The crude incidences of grade III, IV, and V (fatal) rectal complications were 1.1% (17 patients), 1.5% (22 patients), and 0.07% (1 patient), respectively. One patient with a rectal complication died of uncontrolled rectal bleeding 8 years after radiation therapy. The crude incidences of grade III and IV small bowel complications were 0% (0 patients) and 0.7% (10 patients), respectively. The crude incidences of grade III and IV urinary tract complications were 1.5% (23 patients) and 0.8% (12 patients), respectively. The most common grade III complications were haematuria (1.5%) and proctitis (1.1%). The most common grade IV complications were rectovaginal fistula (1.1%) and vesicovaginal fistula (0.8%). Nine patients (0.6%; one with stage I disease, two with stage II disease, five with stage III disease, and one with stage IV disease) required reconstruction of both the urinary tract and lower gastrointestinal tract.

### Second cancers

The incidence of second cancers and observed/expected ratio of incident cancers are shown in Table 3. Second cancers were observed in 13 cases (0.87%). The most frequent sites were the rectum (five cases), colon (three cases), and uterine body (two cases). Analysis on a site-by-site basis revealed an excess of second cancers in the rectum and uterine body and of occurrences of acute leukaemia.

## DISCUSSION

Our radiation therapy regimen differs from the international standard. The concept is unique in that the primary tumour is treated by intracavitary irradiation, and the pelvic and para-aortic lymph nodes are treated by external beam irradiation to a total 50 Gy in 25 fractions. Intracavitary brachytherapy, at a dose of 4 Gy in one fraction routinely prescribed to point A, was performed with two or three insertions per week, for a total of 10 fractions in total. It is essential that both radiation therapies are started simultaneously and that EBRT is performed with a midline shield, no matter what the disease stage. It is difficult to insert the uterine applicator into a bulky tumour at the beginning of treatment. Skill is needed. A midline shield was used throughout the external radiation period, so it is possible that dose of radiation delivered to point A was higher. The other important factor is that the overall treatment time is 7 weeks or less.

Our regimen yielded good results and decreased the incidence of complications for a number of reasons. First, the overall treatment period is short. Several studies have shown that overall treatment time is a significant prognostic factor for patients with cervical cancer treated with radiation therapy alone (Fyles *et al*, 1992; Girinsky *et al*, 1993; Lanciano *et al*, 1993; Perez *et al*, 1995; Delaloye *et al*, 1996). Fyles *et al* (1992) reported the influence of treatment duration on local control. Using three statistical methods of analysis in 830 patients, they observed loss of local control of approximately 1% per day when treatment lasted over 30 days, most evident in stage III and IV patients. Girinsky *et al* (1993) also reported decreased rates of local control and survival when the treatment period was longer than 52 days. By multiple regression analysis, they observed loss of 1.1% local control per day when the treatment period was prolonged from 52 days to more than 62 days. All patients in the current study received radiation therapy within 7 weeks, and this yielded a better result. Second, ICBT is divided into many fractions. According to the linear quadratic model, tumour cells sustain more damage than normal cells by a reduction in the exposure dose and fractionation. The cure rate is improved by controlling normal tissue side effects, easing late complications, and maintaining equal doses of radiation. Intracavitary brachytherapy is more difficult than EBRT, but greater efficacy and fewer complications result (Barendsen, 1982; Fowler, 1989; Brenner and Hall, 1992; Dale and Jones, 1998).

Perez *et al* (1999) investigated correlation between irradiation therapy and sequelae. They graded sequelae as follows: grade 2, producing major symptoms, repeated occurrences requiring short-term (less than 4 weeks) hospitalisation for diagnosis and non-surgical management; grade 3, requiring an operative procedure for correction or prolonged hospitalisation (over 4 weeks) or life threatening. For disease stages II or more, they reported grade 2 morbidity of 10–12% and grade 3 morbidity of 10%. The most frequent grade 2 sequelae were cystitis and proctitis (0.7–3%), and the most common grade 3 sequelae were vesicovaginal fistula (0.6–2%), rectovaginal fistula (0.8–3%), and intestinal obstruction (0.8–4%). Nakano *et al* (2005) also reported late toxicity of radiation therapy. The 10-year actuarial grade 3–5 complication rate was 4.4% in the rectosigmoid colon, 0.9% in the bladder, and 3.3% in the small intestine. Considering these data, morbidity after radiotherapy in our patient population was acceptable. However, survival data of a considerable proportion of the study patients were obtained from the family register database. We believe the survival data are accurate. However, radiotherapy-related morbidity might have been underestimated.

An important issue in the treatment of cervical cancer is how to treat advanced-stage disease, which affects the majority of patients. The reported survival of patients with stage III cervical cancer treated with radiation therapy alone is between 30 and 50% (Barillot *et al*, 1997). Perez *et al* (1999) reported 1456 patients given EBRT (whole pelvis and central shielding, total 50–60 Gy, depending on tumour size) and ICBT (80–90 Gy at point A for stage IIb–IV disease). The 10-year survival rate was 65% for patients with stage IIb disease and 40% for patients with stage III disease, but there were no long-term survivors among patients with stage IV disease. Logsdon and Eifel (1999) reported 983 patients with stage IIIb SCC treated with various radiotherapies, including EBRT and ICBT. The overall survival was 32%. Barillot *et al* (1997) reported a large multi-centre study of 1875 patients treated with radiation alone. Specific survival at 5 years was 70% for stage IIb, 55% for stage IIIa, 45% for stage IIIb, and 10% for stage IV disease. Nakano *et al* (2005) also reported long-term follow-up data for 1148 patients treated with EBRT (whole pelvis and central shielding, total 45–50 Gy at 1.8–2 Gy per fraction) and ICBT (24 Gy in four fractions). The 5- and 10-year cause-specific survival rates were 80 and 74%, respectively, for stage II disease and 66 and 59%, respectively, for stage III disease.

Radiation therapy is known to cause various malignancies, including leukaemia, sarcoma, thyroid carcinoma, and lung carcinoma. Boice *et al* (1985) examined data from 15 cancer registries in eight countries and compared the number of second cancers reported for 182 040 women against the number expected had the same risk prevailed as in the general population. They found an increased risk for cancers of the bladder, rectum, vagina, and caecum. Arai *et al* (1991) reported significantly higher incidences of second cancers in the rectum, bladder, and lung as well as leukaemia. Kleinerman *et al* (1995) described a large-scale study of 49 828 patients with cervical cancer treated with radiation therapy and 16 713 matched patients treated without radiotherapy. They reported that most of the second cancers were of the rectum, vagina, vulva, and bladder, and they concluded that radiation is an important cause of the second cancers, with no evidence that the risk returns to a normal level. Second cancers were observed in 13 of our patients (0.87%), most frequently in the rectum (five cases), colon (three cases), and uterine body (two cases). Although there are many reports on radiation-induced cancer, such cancers occurred in less than 1% of our patients. Although our radiotherapy regimen causes some complications, the benefits of the treatment outweigh the disadvantages. Continued improvement in radiotherapeutic techniques, along with diagnosis at younger ages and earlier stages, will result in longer survival times for patients. This may in turn increase the significance of radiation-related second cancers.

In recent years, several groups have reported concurrent chemoradiation to improve the survival of patients with locally advanced cervical carcinoma (Keys *et al*, 1999; Morris *et al*, 1999; Rose *et al*, 1999; Whitney *et al*, 1999; Peters *et al*, 2000). Cisplatin is the most active cytotoxic agent against cervical cancer. Questions pertaining to treatment of cervical cancer are focused mainly on chemotherapy regimens, so there is a tendency to ignore the radiotherapy method.

In 1999, the National Cancer Institute (USA) published an announcement stating that cisplatin-based chemotherapy should be used concomitantly with radiation therapy in cases of cervical cancer. However, there were not an adequate numbers of patients with advanced cancer in the studies published, particularly patients with pelvic and/or para-aortic lymph node metastasis. We know from surgical series that the incidence of positive para-aortic nodes is less than 10% for stage II, 20% for stage II, 30% for stage III, and 40% for stage IV disease (Berman *et al*, 1984). Although the current study is a retrospective one, it involved a purely consecutive series of patients regardless of pelvic and/or para-aortic lymph node status before treatment, so the data may be of great value.

In conclusion, long-term results of our ICBT/EBRT regimen for cervical cancer are reviewed herein. Our method of irradiation is unique, but it provides a good result and a decreased incidence of complications. Our study is one of only a few long-term follow-up studies involving a large number of patients, and it yielded valuable data pertaining to the incidence of second cancers following radiation therapy for cervical cancer. The standard treatment for locally advanced cervical cancer is gradually changing to concurrent chemoradiation. The main issue in the treatment of cervical cancer is how chemotherapy is used, but we believe the radiation methodology needs further discussion.

## Figures and Tables

**Table 1:**
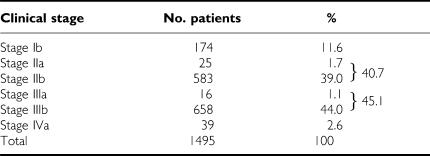
Cervical carcinomas treated with radiotherapy alone

**Table 2:** Grades of late complications according to site

	**Grade III**	**Grade IV**	**Grade V (fatal)**
Rectum	13 (0.9%)	21 (1.4%)	1 (0.07%)
Small bowel	11 (0.7%)	—	—
Bladder	17 (1.1%)	9 (0.6%)	—
Combined	—	10 (0.7%)	—
Total	41 (2.7%)	40 (2.7%)	1 (0.07%)

Total 82 cases (5.5%).

**Table 3:** Second cancers after treatment with radiotherapy alone

**Site**	**Observed**	**Expected**	**O/E**
Rectum	5	1.9	2.6
Colon	3	3.3	0.9
Uterine body	2	0.7	2.9
Ovary	1	1.0	1.0
Acute leukaemia	1	0.5	1.9
Pelvic MFH	1	—	—

Abbreviation: MFH=malignant fibrous histiocytoma.
